# A surgical case of abscess of the sternal region due to synovitis-acne-pustulosis-hyperostosis-osteitis (SAPHO) syndrome

**DOI:** 10.1186/s44215-023-00093-8

**Published:** 2023-08-01

**Authors:** Makoto Tada, Hirofumi Uehara, Takeshi Ohyu, Atsushi Watanabe

**Affiliations:** 1 Department of Thoracic Surgery, Hakodate Goryoukaku Hospital, 38-3 Goryokakucho, Hakodate, Hokkaido Japan; 2 Department of Thoracic Surgery, Sapporo Medical University School of Medicine and Hospital, South 1, West 16, Chuo-Ku, Sapporo, Hokkaido Japan

**Keywords:** Synovitis-acne-pustulosis-hyperostosis-osteitis (SAPHO) syndrome, Sternoclavicular joint abscess, Negative pressure wound therapy (NPWT)

## Abstract

**Background:**

Synovitis-acne-pustulosis-hyperostosis-osteitis (SAPHO) syndrome is a rare chronic inflammatory disorder characterized by cystic skin disease, aseptic osteomyelitis, and hyperostosis. It is rarely associated with subcutaneous abscesses in the anterior chest wall. Herein, we describe a case of a sternal abscess due to SAPHO syndrome that was successfully treated with debridement.

**Case presentation:**

A 62-year-old female patient presented with a chief complaint of a mass in the right anterior chest region. Computed tomography showed the presence of an abscess around the sternum. Magnetic resonance imaging revealed inflammation of the spine. The patient also had a history of palmoplantar pustulosis, raising the suspicion of SAPHO syndrome; however, sternoclavicular joint infection could not be excluded. Abscess drainage and wall debridement were performed. However, reaccumulation of the abscess occurred early after surgery. Although culture tests of the abscess were negative, histopathological examination of the abscess wall revealed a tuberculoid granuloma. Owing to unsuccessful open wound management, debridement of the anterior thoracic skin and abscess wall and partial sternal resection were performed 2 months after the initial surgery. Negative-pressure wound therapy (NPWT) was applied for 4 weeks after surgery, followed by immunosuppressant administration. The patient underwent split-thickness skin grafting on postoperative day 65 and had no abscess recurrence at 18 months post-surgery.

**Conclusions:**

Abscesses in the sternal region caused by SAPHO syndrome can be difficult to differentiate from infectious arthritis. Therefore, drainage and debridement should be considered if infection cannot be excluded.

## Background

Synovitis-acne-pustulosis-hyperostosis-osteitis (SAPHO) syndrome is a rare chronic inflammatory disorder characterized by palmoplantar pustulosis and other cystic skin diseases, aseptic osteomyelitis, and hyperostosis, and was proposed by Chamot et al. in 1987 [1]. The exact etiology of this disease remains unknown, and diagnostic criteria and treatment strategies have not been established. SAPHO syndrome may be associated with abscesses in the sternal region; however, there are few reports on its treatment.

## Case presentation

**Fig. 1:**
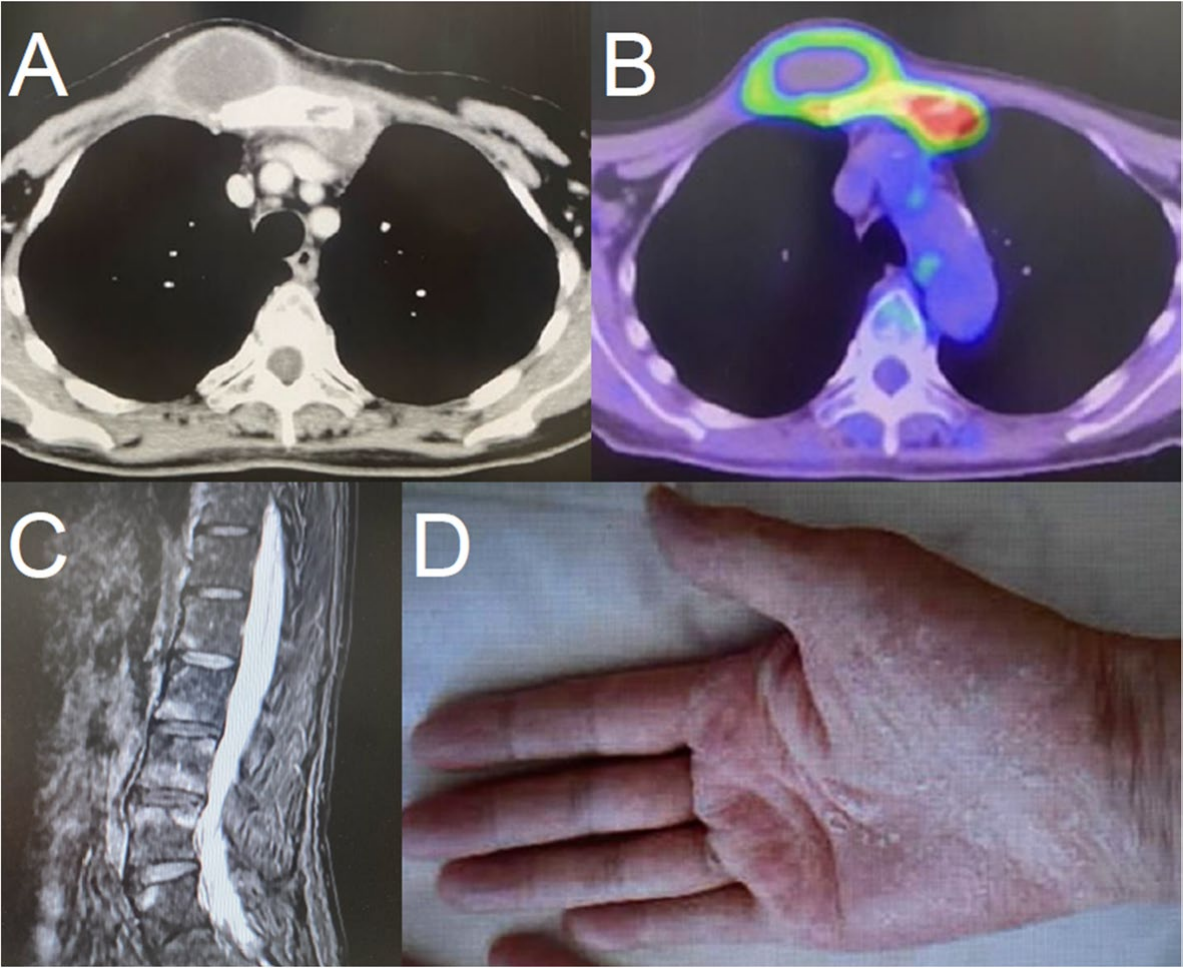
**A**, **B** CT and 18F-FDG PET/CT showing inflammatory cystic lesions measuring 49 × 45 × 27 mm at the right sternoclavicular joint and 26 × 17 × 9 mm at the mediastinal side of the left first rib joint. **C**, **D** Characteristic findings of SAPHO syndrome. **C** MRI showing patchy long T2 signals along the lumbar vertebrae. **D** Image showing palmoplantar pustulosis. CT, computed tomography; 18F-FDG PET/CT,18-fluorine-fluorodeoxyglucose positron-emission tomography/computed tomography; MRI, magnetic resonance imaging; SAPHO: synovitis-acne-pustulosis-hyperostosis-osteitis

A 62-year-old female patient with no comorbidities presented with a chief complaint of a right anterior chest mass. Computed tomography and 18-fluorine-fluorodeoxyglucose positron-emission tomography/computed tomography revealed inflammatory cystic lesions at the right sternoclavicular joint and mediastinal side of the left first rib joint (Fig. 1A, B). Apart from tenderness, the patient was asymptomatic, and inflammatory markers were within normal limits. SAPHO syndrome was suspected based on the presence of vertebral inflammation on magnetic resonance imaging (Fig. 1C) and a history of palmoplantar pustulosis (Fig. 1D). Infectious arthritis of the sternoclavicular joint could not be completely ruled out, and the lesion was in contact with the left brachiocephalic vein, raising concerns regarding perforation. Therefore, abscess drainage and abscess wall debridement were performed for diagnostic and therapeutic purposes. Yellowish pus was drained, and a drainage tube was placed behind the sternum. The patient was administered SBT/ABPC for 10 days following the surgery. The drainage tube was removed on postoperative day (POD) 5; however, re-accumulation of the abscess occurred 1 week later. Although culture tests of the abscess were negative, histopathological examination of the abscess wall revealed tuberculoid granulomas, and malignancy was excluded. The IGRA test and PCR tests of the abscess for Mycobacterium tuberculosis yielded negative results. Histologically, the abscess wall exhibited tuberculoid granulomas with neutrophilic infiltration, which was contiguous with sternum osteomyelitis. This finding could potentially be interpreted as a sterile abscess associated with SAPHO syndrome. However, since a tuberculoid granuloma is not typically observed in SAPHO syndrome, the use of steroids and immunotherapy was not recommended.

**Fig. 2:**
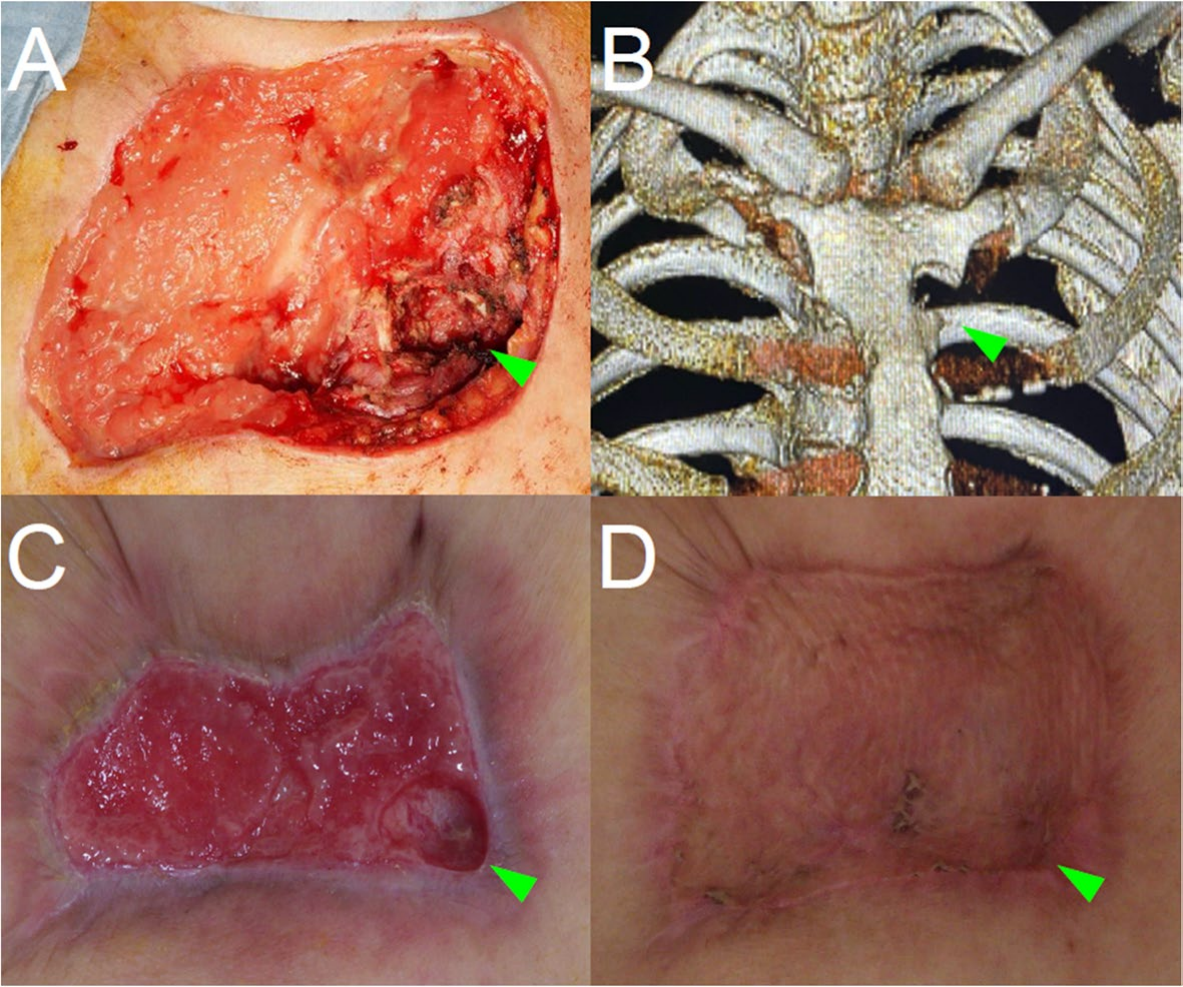
**A**, **B** Intraoperative findings and CT showing the debridement of the anterior thoracic skin and abscess wall and partial sternal resection (arrowheads showing the area of partial sternum resection). **C** Image showing open wound in which abscess formation was controlled by 4 weeks of NPWT. **D** Image showing wound after split-thickness skin grafting. CT: computed tomography, NPWT: negative pressure wound therapy

Despite open wound management to control the abscess, no improvement was observed. Therefore, 2 months after the initial surgery, debridement of the anterior thoracic skin and abscess wall, and partial sternal resection were performed at the level of the left first intercostal space to expose the abscess cavity behind the sternum (Fig. 2A, B). The patient was administered CEZ for 19 days after the second surgery. Negative pressure wound therapy (NPWT) was administered for 4 weeks starting on POD 5. Although SAPHO syndrome was still the primary diagnosis, immunosuppressive therapy was withheld because of the possibility of nontuberculous mycobacterial (NTM) infection based on the granulomatous inflammation observed in the abscess wall. During the clinical course following the initial visit, the blood laboratory findings consistently remained within the normal range, with the exception of elevated white blood cell counts and C-reactive protein levels observed during the acute postoperative period. Immunosuppressive therapy with tacrolimus 3 mg daily was initiated on POD 35 when the abscess was adequately controlled (Fig. 2C). The patient underwent split-thickness skin grafting on POD 65, and there was no recurrence of the abscess at 18 months post-surgery (Fig. 2D).

## Discussion and conclusions

The SAPHO syndrome is characterized by synovitis, acne, pustulosis, hyperostosis, and osteitis [1]. Among these, aseptic osteitis and osteomyelitis of the sternoclavicular joint are considered the core symptoms [2], and there have been reports of sternal and sternoclavicular joint abscesses secondary to these conditions [3]. However, such reports are limited, and SAPHO syndrome is generally not well recognized by thoracic surgeons. This case report highlights the significance of considering SAPHO syndrome as a potential etiology of sternal abscesses that can be difficult to differentiate from infectious arthritis. The diagnostic and therapeutic challenges encountered in this case may be informative for thoracic surgeons managing similar cases, emphasizing the need for a multidisciplinary approach and circumspect consideration of the differential diagnoses. Therefore, this case report may assist thoracic surgeons in the differential diagnosis and treatment of similar abscesses in the sternal region.

Currently, no specific clinical features or laboratory findings have been established to confirm a diagnosis of SAPHO syndrome. It is primarily diagnosed by exclusion based on clinical and radiological symptoms [2]. Although the abscess formed is usually sterile, it is essential to exclude infectious arthritis and tumor conditions of the bone according to the diagnostic criteria proposed by Kahn [4]. Therefore, active drainage and debridement, with tissue sampling for histopathological and culture tests, should be considered in cases of abscess formation.

Nonsteroidal anti-inflammatory drugs are typically used as the first-line therapy for SAPHO syndrome; however, in most cases, they do not provide sufficient therapeutic effects on their own, and immunosuppressants, such as corticosteroids, methotrexate, sulfasalazine, and cyclosporine, are often used. It is often difficult to rule out the possibility of infectious arthritis of the sternoclavicular joint when examining a patient with an anterior chest wall abscess. Thus, immunosuppressive drugs must be cautiously administered. There have been cases in which patients were misdiagnosed with SAPHO syndrome instead of infectious arthritis of the sternoclavicular joint, resulting in increased severity of the condition with the administration of immunosuppressive drugs [5]. Although SAPHO syndrome was initially suspected in this case, it was difficult to exclude the possibility of NTM infection because of the granulomatous inflammation of the abscess wall. Therefore, tacrolimus was cautiously initiated only after the abscess was adequately controlled by debridement with partial sternal resection and NPWT of the open wound, and cultures for nontuberculous mycobacteria were determined to be negative.

When examining an abscess in the sternal region, surgeons should always consider SAPHO syndrome as a differential diagnosis, in addition to infectious arthritis and tumoral conditions. Abscesses in the sternal region caused by SAPHO syndrome can be difficult to distinguish from infectious arthritis. Although the diagnostic process and treatment strategies may be controversial, drainage and debridement should be considered if infection cannot be excluded, and immunosuppressive drugs should be cautiously administered.

## Data Availability

All data from this study are presented in the manuscript. Some datasets are available from the corresponding authors upon request.

## Abbreviations

SAPHO: Synovitis-acne-pustulosis-hyperostosis-osteitis
POD: Postoperative day
NPWT: Negative pressure wound therapy
NTM: Nontuberculous mycobacterial

## References

[1] Chamot AM, Benhamou CL, Kahn MF, Beraneck L, Kaplan G, Prost A. Acne-pustulosis-hyperostosis-osteitis syndrome. Results of a national survey. 85 cases. Rev Rhum Mal Osteoartic. 1987;54:187–96. 2954204

[2] Liu S, Tang M, Cao Y, Li C. Synovitis, acne, pustulosis, hyperostosis, and osteitis syndrome: review and update. Ther Adv Musculoskelet Dis. 2020;12:1759720X20912865. 32523634 10.1177/1759720X20912865 PMC7236399

[3] Schwartz GS, Rios L, Zivin-Tutela T, Bhora FY, Connery CP. Uncommon etiology of an anterior chest wall mass. Ann Thorac Surg. 2009;88:e58–9. 19853079 10.1016/j.athoracsur.2009.07.090

[4] Kahn MF. Proposed classification criteria of SAPHO syndrome. American college of rheumatology 67th Annual Scientific Meeting. 2003.

[5] Wako Y, Sakamoto M, Rokkaku T, Motegi H, Watanabe H, Yamada T, et al. A case of hip joint septic arthritis due to haematogenous infection, which was misdiagnosed sternoclavicular joint septic arthritis as SAPHO syndrome. Mod Rheumatol Case Rep. 2021;5:409–13. 33427583 10.1080/24725625.2020.1869510

